# Supramolecular chemistry under mechanochemical conditions: a small molecule template generated and integrated into a molecular-to-supramolecular and back-to-molecular cascade reaction†

**DOI:** 10.1039/c9sc05823k

**Published:** 2020-03-10

**Authors:** Shweta P. Yelgaonkar, Dale C. Swenson, Leonard R. MacGillivray

**Affiliations:** Department of Chemistry, University of Iowa Iowa City IA 52242 USA len-macgillivray@uiowa.edu

## Abstract

We describe the integration of a small-molecule hydrogen-bond-donor template into a cascade reaction that is comprised of a combination of molecular and supramolecular events. The cascade is performed mechanochemically and in the presence of μL amounts of water. The small-molecule template is generated (molecular) using water-assisted vortex grinding and is then used to assemble an alkene (supramolecular) to undergo an intermolecular [2 + 2] photodimerization reaction (molecular). The chemical cascade results in a cyclobutane photoproduct that we show serves as a building block of a hydrogen-bonded network with a topology that conforms to T-silica. Remarkably, the molecular–supramolecular–molecular chemical cascade occurs stepwise and entirely regioselectively within the continuous mechanochemical conditions employed.

## Abstract 2

Mechanochemistry is applied to molecular and supramolecular chemistry to support a template-directed photochemical reaction.

## Graphical abstract

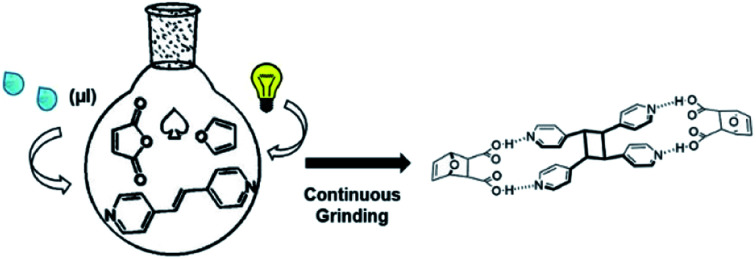

## Introduction

Cascade reactions are of great interest in organic synthetic chemistry and, more recently, biotechnology.^1–4^ Cascade reactions are critical for biological process. That two or more reactions can be developed to operate in sequence has inspired chemists to create cascades that generate products (*e.g.* natural products) of considerable complexity. Cascade processes in biotechnology harness molecular recognition capabilities of enzymes to convert substrates to enzymatic reactions.^5,6^ Many current efforts in the field of molecular recognition vis-à-vis supramolecular chemistry focus to develop molecules that direct covalent bond formation.^7,8^ Such molecules, or templates, assemble reactants, similar to enzymes, by noncovalent forces (*e.g.* hydrogen bonds) to undergo intra- and intermolecular covalent-bond-forming reactions. Whereas natural enzymes are now more regularly integrated into cascade processes, and while organic catalysts are now playing prominent roles in related organocascades,^9,10^ there have been no reports wherein a small organic molecule that functions as a template has been integrated into a cascade process.

**Scheme 1:**
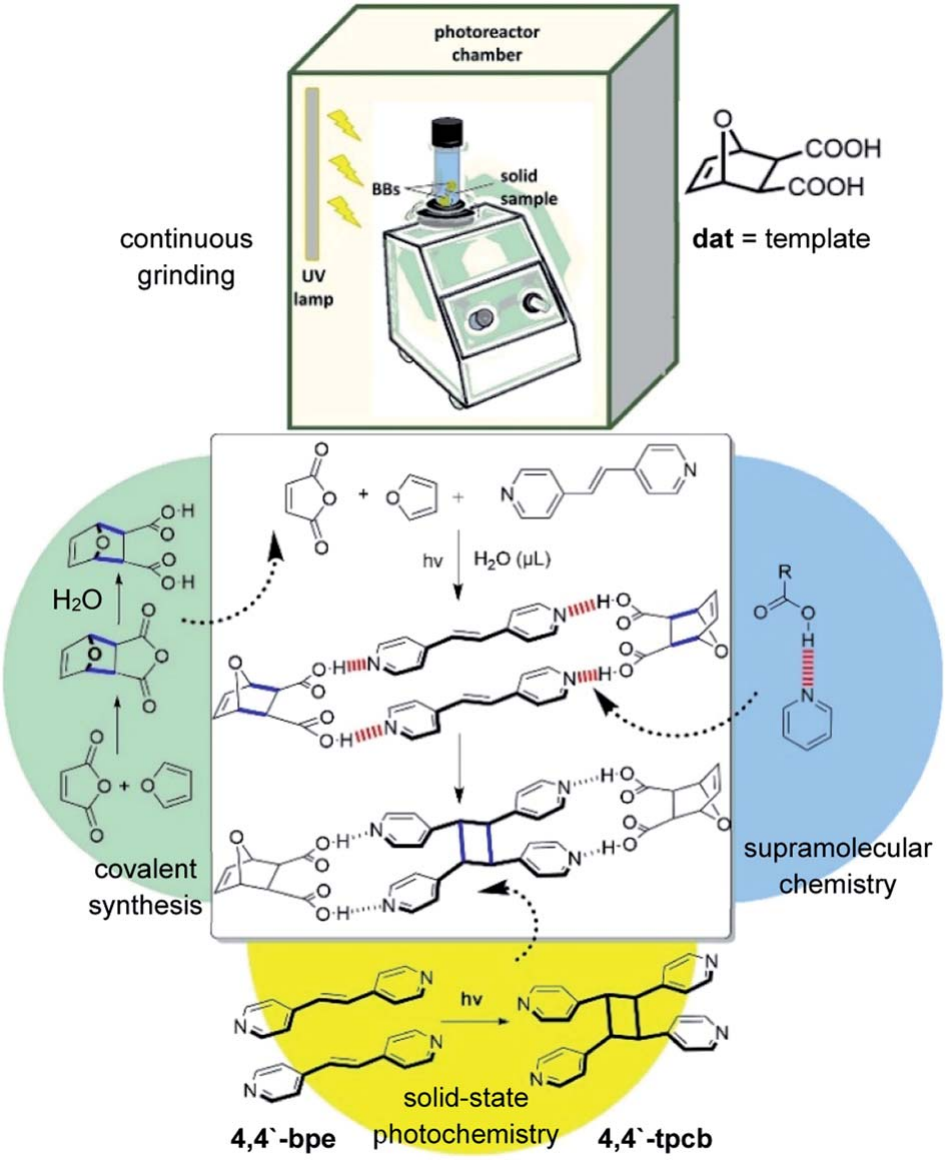
Mechanochemical cascade with supramolecular template **dat** that generates **4,4′-tpcb** with vortex grinding.

Here, we describe the integration of a small ditopic template molecule into a one-pot cascade reaction (Scheme 1). The cascade is achieved mechanochemically^11–13^ and *via* the solid state wherein a small-molecule template itself and a photochemically reactive self-assembled hydrogen-bonded complex are formed by vortex grinding.^14^ The cascade is comprised of a Diels–Alder reaction that generates the diacid template **dat**. The template then assembles the alkene **4,4′-bpe** in a four-component supramolecular assembly in the cocrystal 2(**dat**)·2(**4,4′-bpe**) wherein **bpe** is positioned by the template to undergo a [2 + 2] photocycloaddition reaction. Continuous grinding and application of ultraviolet (UV) light afford **4,4′-tpcb** stereoselectively and in quantitative yield. We consider our results an important first step towards integrating covalent-bond-forming reactions mediated by principles of supramolecular chemistry into cascade processes.

## Results and discussion

**Scheme 2:**
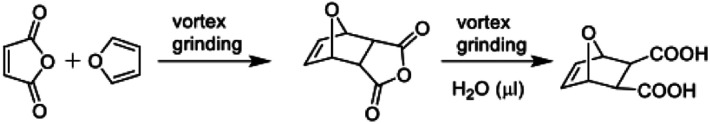
Mechanochemical synthesis of template **dat**.

The small-molecule template used in the cascade process is generated by a Diels–Alder reaction of maleic anhydride and furan (Scheme 2). That the Diels–Alder reaction can occur mechanochemically while rare has been reported,^15^ with the formation of relatively simple bicyclic and more complex (*e.g.* iptycenes) products being recently described.^16,17^ We envisaged that the cisoid disposition of the two acid groups of **dat** could be used to assemble **bpe** in a solid^18–20^*via* hydrogen bonds (*i.e.* C=C bonds separated <4.2 Å and parallel) for an intermolecular [2 + 2] photodimerization to give **4,4′-tpcb**. The cascade would be set in motion with Diels–Alder generation of **dat** (molecular). A subsequent cocrystallization of **dat** and **bpe** would then preorganize **bpe** by hydrogen bonds (supramolecular) for a [2 + 2] photodimerization (molecular). In doing so, the bicyclic structure of **dat** would be generated *in situ* and the newly-formed acid groups would participate in hydrogen bonds with the pyridyls of the alkene.

To date, cascade reactions that integrate both principles of supramolecular chemistry and are performed mechanochemically remain largely unexplored.^21^ Examples of cascades that incorporate principles of supramolecular chemistry include the generation^22,23^ and post-modification^22^ of coordination cages. The examples were performed in solution. Examples of cascades that involve principles of supramolecular chemistry and are performed mechanochemically involve metal coordination that generates a halogen-bonded metal–organic solid.^24^ An organic approach based on salt generation followed by a photodimerization in the solid state has also been described.^25^ We note that there have been many examples of multicomponent reactions performed mechanochemically yet in the absence of supramolecular processes.^21^

### Mechanochemical generation of template

In initial experiments, we determined that **dat** forms by Diels–Alder reaction mechanochemically with vortex grinding^14^ and in the presence of a small amount of water. When a mixture of maleic anhydride (49 mg, 0.5 mmol) and furan (40.8 mg, 0.6 mmol) was subjected to vortex grinding (premium grade steel balls, 5 mm diameter) for a period of 1 h and in the absence of water, only the thermodynamically favored *exo*-anhydride **dat-anh** formed in quantitative yield. The formation of **dat-anh** was monitored using ^1^H NMR spectroscopy (10 min intervals). The diacid **dat**, however, formed quantitatively using liquid-assisted grinding (LAG)^26^ for a period of 1 h in the presence of a small amount (40 μL) of water. The generation of the acid groups is attributed to hydrolysis of **dat-anh**. The *exo*-stereochemistry of **dat** was elucidated by a singlet at 2.63 ppm.

A single-crystal X-ray structure analysis confirmed the *exo*-stereochemistry of **dat**. While **dat** was originally described nearly five decades ago, a crystal structure had not been reported.^27^ Colorless single crystals as prisms of **dat** were obtained by slow evaporation (10 mg) of a saturated ethyl acetate solution (5 mL) over a period of 2 d.

**Fig. 1:**
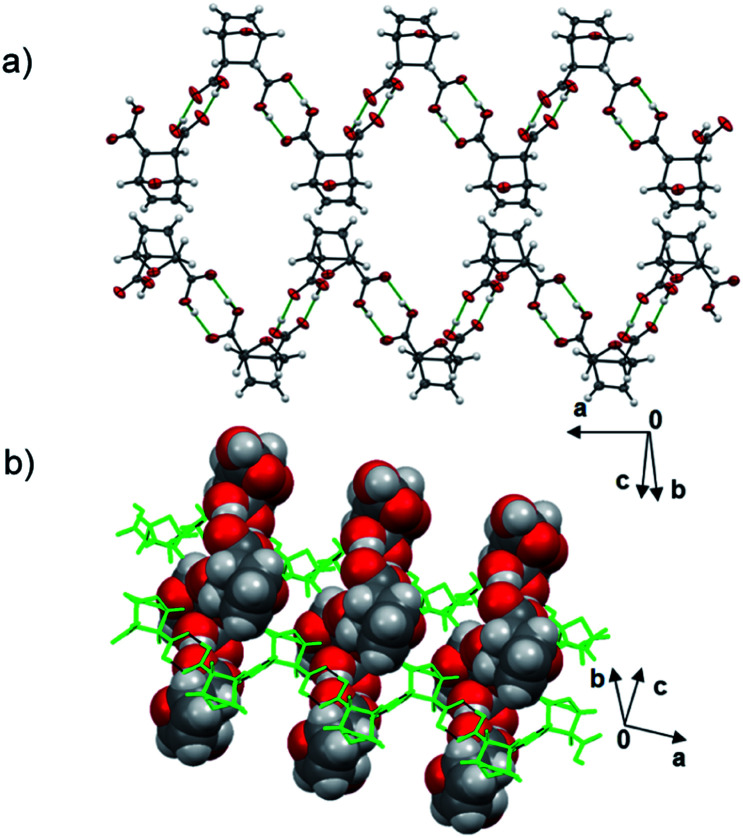
X-ray structure **dat**: (a) hydrogen-bonded dimer and (b) zigzag chains.

**Dat** crystallizes as a pure form in the chiral monoclinic space group *P*2_1_ with eight molecules in the asymmetric unit. The diacid self-assembles *via* hydrogen-bonded carboxylic acid dimers [O⋯O distance range (Å) 2.593(1)–2.737(1)] as one-dimensional (1D) zigzag chains that run approximately orthogonal (Fig. 1). Six of the eight molecules lie disordered in terms of either the bicyclic core (4 total) or the –CO_2_H groups (2 total).

### Templated photodimerization

**Scheme 3:**
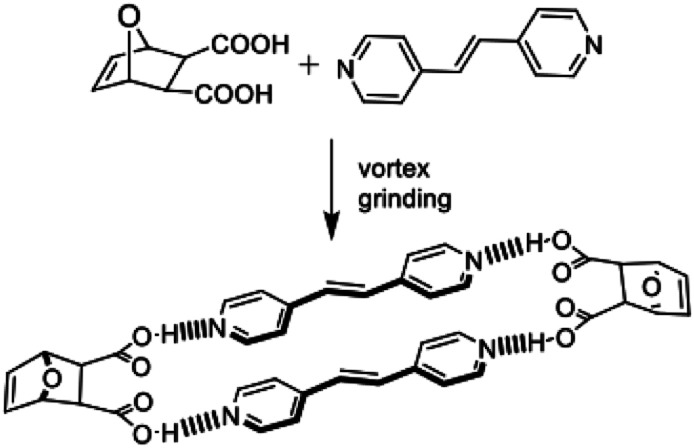
Mechanochemical synthesis of 2(**dat**)·2(**4,4′-bpe**).

**Dat** acts as a template of the [2 + 2] photodimerization in the crystalline state. Specifically, application of vortex grinding to a mixture of **dat** and **4,4′-bpe** (1 : 1 ratio) for 40 min afforded a homogeneous off-white powder (Scheme 3). Analysis by powder X-ray diffraction (PXRD) revealed a phase with prominent peaks at 2*θ* = 17.8, 25.3, 26.6 and 27.8°. When the powder was subjected to UV-radiation (450 W medium pressure Hg vapor lamp) for 25 h, **4,4′-tpcb** formed quantitatively as confirmed by ^1^H NMR spectroscopy (**DMSO-d6**).

A single-crystal X-ray diffraction study confirmed **dat** to act as a template of the photoreaction. When the initially ground powder (20 mg) was recrystallized in acetonitrile/methanol (v/v 1 : 1), colorless blade-like crystals of 2(**dat**)·2(**4,4′-bpe**) suitable for X-ray analysis formed upon slow evaporation.

**Fig. 2:**
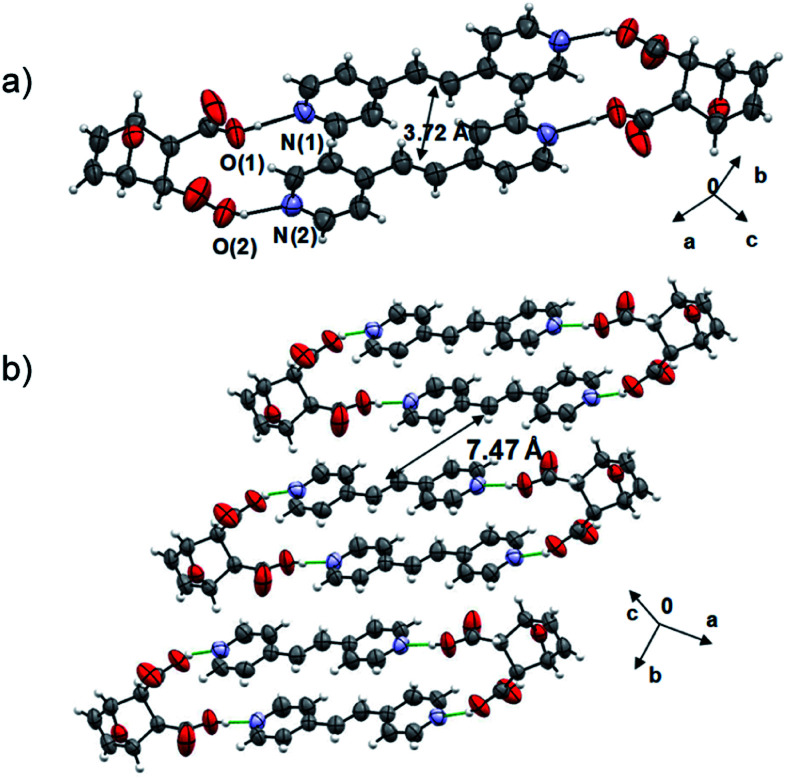
X-ray structure 2(**dat**)·2(**4,4′-bpe**): (a) hydrogen-bonded assembly and (b) face-to-face stacks.

The components of 2(**dat**)·2(**4,4′-bpe**) crystallize in the triclinic space group *P*1̄. The molecules form a discrete four-component assembly sustained by four O–H⋯N hydrogen bonds [O⋯N separations (Å): O(1)⋯N(1) 2.644(3), O(2)⋯N(2) 2.640(3)] (Fig. 2), with the carbon–carbon double (C=C) bonds parallel and stacked in close proximity (3.72 Å). The geometry conforms to the criteria of Schmidt for a photoreaction.^28^ Olefins of neighboring assemblies lie stacked face-to-face and offset along the *b*-axis and outside the distance criterion (7.47 Å). We are unaware of an example of a product of a Diels–Alder reaction acting as a template of a photodimerization.

### Cascade under mechanochemical conditions

**Fig. 3:**
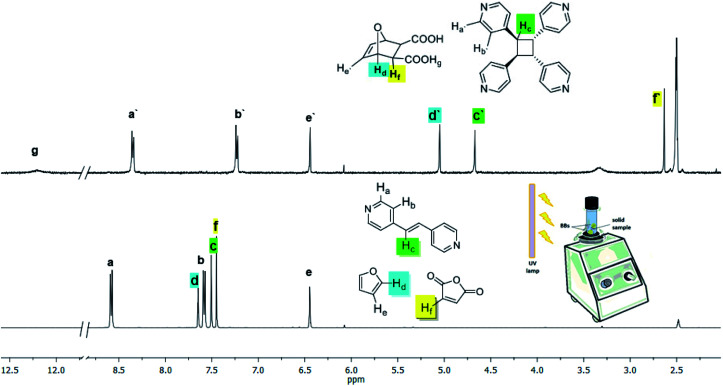
^1^H NMR spectra before (bottom) and after (top) vortex grinding of maleic anhydride, furan, and **4,4′-bpe** with application of UV light and in presence of water (NMR solvent: **DMSO-d6**).

The Diels–Alder reaction to form **dat**, the cocrystallization to form 2(**dat**)·2(**4,4′-bpe**), and the photodimerization to generate **4,4′-tpcb** all occur in a mechanochemically-mediated cascade process. Specifically, vortex grinding of a mixture of maleic anhydride, furan, and **4,4′-bpe** – in the absence of both UV-light and water – for 25 h afforded a mixture of **dat** (7% yield) and **dat-anh** (93% yield). Vortex grinding of the same components with UV-light yet in the absence of water for 25 h afforded **4,4′-tpcb** stereoselectively and albeit in low yield (5% yield). We note that both **dat** (65% yield) and **dat-anh** (35% yield) formed in the vortex grinding process. Moreover, we determined that the same reaction conditions using UV-radiation and without water generates **dat-anh** quantitatively in 1 h. When 40 μL of water is introduced to the mixture following the initial 1 h of grinding, **4,4′-tpcb** formed stereoselectively and in quantitative yield with grinding in 10 h (Fig. 3). We conclude that the presence of water accelerates the formation of **dat** and the photoactive cocrystal 2(**dat**)·2(**4,4′-bpe**) upon vortex grinding conditions. We note that the resulting solid is crystalline by PXRD. **4,4′-bpe** does not act as a dienophile under the grinding, which is consistent with solution studies.^29^

### T-silica topology

A capacity of the Diels–Alder product **dat** to interact with photochemical product **4,4′-tpcb** was determined by single-crystal X-ray diffraction. When a sample of the photoreacted solid (20.0 mg) subjected to the vortex grinding was recrystallized by slow evaporation from THF–methanol (4 : 1 v/v) single crystals of 2(**dat**)·(**4,4′-tpcb**)·**THF** as plates formed.

**Fig. 4:**
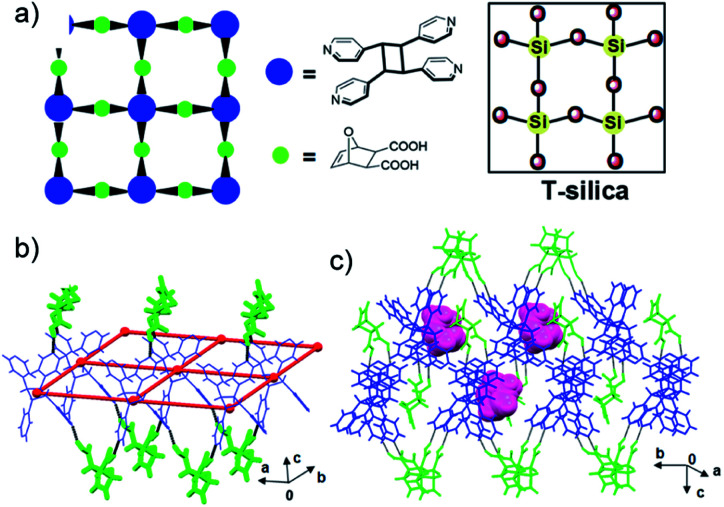
X-ray structure 2(**dat**)·(**4,4′-tpcb**)·THF: (a) topology of 2D grid that conforms to T-silica, (b) **dat** (green) as angular bridges, and (c) THF (pink) in cavities between stacked grids.

The components of 2(**dat**)·(**4,4′-tpcb**)·**THF** crystallize in the chiral orthorhombic space group *P*2_1_2_1_2_1_. The diacid and tetrapyridine assemble *via* O–H⋯N hydrogen bonds [O⋯N separation (Å): O(1)⋯N(1) 2.677(1), O(2)⋯N(2) 2.673(1)] to form a 2D hydrogen-bonded framework that conforms to a square grid (sql) topology (Fig. 4).^30^ In the grid, **4,4′-tpcb** and **dat** function as tetrahedral nodes (cyclobutane: angles 109.1123.4°; separations: 10.9 × 11.4 Å) and angular bridges (72.0°), respectively. The corner acute angles supplied by **dat** occur along lines of common edges and alternate above and below each grid. The grids stack to form cavities occupied by THF molecules. The angular nature of the bridges means that the topology of the grid 2(**dat**)·(**4,4′-tpcb**) conforms to T-silica, which is a recently calculated and metastable form of SiO_2_.^31^ We are unaware of a molecular network that conforms to the topology of T-silica. We note that there is no match between the simulated X-ray pattern of 2(**dat**)·(**4,4′-tpcb**)·**THF** and the powder immediately following the mechanical cascade.

## Conclusions

In conclusion, we have utilized the product of a Diels–Alder reaction as a template of a [2 + 2] photodimerization. The template was generated mechanochemically and directs a [2 + 2] photodimerization of **4,4′-bpe** to give **4,4′-tpcb** stereoselectively and in quantitative yield in a cascade process. A templated solid–state reaction has, thus, been integrated into a mechanochemically-mediated cascade reaction using vortex grinding. We now focus to broaden the scope of the mechanochemical and self-assembly processes involving additional concepts of supramolecular chemistry and mechanochemistry.

### Experimental procedure

In typical procedure, maleic anhydride (49 mg, 0.5 mmol), furan (40.8 mg, 0.6 mmol), and **4,4′-bpe** (91 mg, 0.5 mmol) together with two metal balls (premium grade steel 420 (hard)) of 5 mm diameter, were introduced into an ACE pressure tube. The tube was mounted onto the vortex mixer and secured with a clamp to a stand rod. Vortex set up was placed in the photoreactor with a broadband UV lamp. The mixer was ground for 1 h and progress of adduct formation was monitored using ^1^H NMR spectroscopy. After addition of water, the powdered sample was simultaneously ground in the vortex apparatus and irradiated (450 W medium-pressure mercury lamp). The formation of the photoproduct was monitored by ^1^H NMR spectroscopy (see, also: ESI†).

### Single-crystal X-ray diffraction

Single-crystal diffraction data were collected on a Nonius Kappa CCD or Nonius APEX II Kappa single-crystal X-ray diffractometer using MoKα radiation (*λ* = 0.71073 Å). Structure solution and refinement were accomplished using Olex2,^32^ SHELXT^33^ and SHELXL.^34^ All nonhydrogen atoms were identified from the difference Fourier map within several refinement steps. Hydrogen atoms associated with carbon atoms were refined in geometrically constrained positions.

Crystal data for **dat** (CCDC 1876029): C_8_H_8_O_5_*M* = 184.14 g mol^−1^: monoclinic, *P*2_1_, *a* = 9.2321(19) Å, *b* = 18.768(4) Å, *c* = 18.876(4) Å, *β* = 93.72(3)°, *V* = 3263.8(11) Å^3^, *Z* = 16, *T* = 190(2) K, μ(MoKα) = 0.127 mm^−1^, 50 626 reflections measured, 12 483 unique (*R*_int_ = 0.0553), *R*_1_(obs) = 0.0584, w*R*_2_(all) = 0.1632.

Crystal data for 2(**dat**)·2(**4,4′-bpe**) (CCDC 1876028): C_20_H_18_N_2_O_5_*M* = 366.36 g mol^−1^, triclinic, *P*1̄, *a* = 8.2700(8) Å, *b* = 9.1848(9) Å, *c* = 12.4194(12) Å, *α* = 71.619(5)°, *β* = 82.065(5)°, *γ* = 79.191(5)°, *V* = 876.19(15) Å^3^, *Z* = 2, *T* = 298(2) K, μ(MoKα) = 0.101 mm^−1^, 5206 reflections measured, 3148 unique (*R*_int_ = 0.0166), *R*_1_(obs) = 0.0604, w*R*_2_(all) = 0.1734.

Crystal data for 2(**dat**)·(**4,4′-tpcb**)·THF (CCDC 1895859): C_44_H_44_N_4_O_11_*M* = 804.83 g mol^−1^: orthorhombic, *P*2_1_2_1_2_1_, *a* = 10.9162(11) Å, *b* = 11.3807(11) Å, *c* = 31.809(3) Å, *V* = 3951.8(7) Å^3^*, Z* = 4*, T* = 150(2) K, μ(MoKα) = 0.098 mm^−1^, 40 673 reflections measured, 6949 unique (*R*_int_ = 0.0491), *R*_1_(obs) = 0.0591, w*R*_2_(all) = 0.1643.

## Conflicts of interest

There are no conflicts to declare.

## Supplementary Material

SC-011-C9SC05823K-s001
